# Evaluation of Race as a Predictor of Fear of Falling in Black Older Adults

**DOI:** 10.1093/geroni/igaa057.757

**Published:** 2020-12-16

**Authors:** Selena Washington, Susan Stark, Makenna Synder, Yi-Ling Hu, Brittany Minor

**Affiliations:** 1 Saint Louis University, Saint Louis, Missouri, United States; 2 Washington University in St. Louis, St. Louis, Missouri, United States; 3 Washington University, St. Louis, Missouri, United States; 4 Wayne State University, Detroit, Michigan, United States

## Abstract

Fear of falling (FOF) is a common issue and health concern reported by adults 65 years of age and older.There is mixed evidence about potential disparity in fall incident rates due to race and FOF, and it is unclear if and under what circumstance falls rates and risks differ by racial ethnicity. The purpose of this study is to determine if race predicts fear of falling in older adults at greater risk for falls; and to examine the relationship between activities of daily living (ADL) and mobility performance measures with FOF. A cross-sectional observational study was used to examine predictors of FOF among community dwelling-older adults using data from two longitudinal randomized clinical trials (RCT). Participants (N=259) had a mean age of 75.7 ±7.4, 78.8% female, 80.64% Black, and 77.03% White or Other, with ≥ 1 fall in the last 12 months. Subjects completed a demographic profile; the Tinetti and Short Falls Efficacy Scale to assess FOF; and activities of daily living (ADL) and mobility performance scales to assess function. The chi square analysis revealed Black older adults were two times more likely to report FOF (OR = 2.17, 95% CI = 1.14, 4.15; p=.05) in comparison to White older adults. The regression analysis demonstrated that race is a significant factor to predict FOF (OR = .67, 95% CI = 1.21, 3.24; p=.05); and the descriptive analysis revealed significantly worse ADL and mobility function scores within the high FOF group.

